# Developing national cancer survivorship standards to inform quality of care in the United States using a consensus approach

**DOI:** 10.1007/s11764-024-01602-6

**Published:** 2024-05-13

**Authors:** Michelle A. Mollica, Gina McWhirter, Emily Tonorezos, Joshua Fenderson, David R. Freyer, Michael Jefford, Christopher J. Luevano, Timothy Mullett, Shelley Fuld Nasso, Ethan Schilling, Vida Almario Passero, the National Cancer Survivorship Standards Subject Matter Expert Group, Catherine Alfano, Precilla Belin, Anne Blaes, Hillary Cavanagh, Lanie Francis, David R. Freyer, Danielle Friedman, Shelley Fuld Nasso, Patricia Ganz, Min He, Batsheva Honig, Shawna Hudson, Linda Jacobs, Michael Jefford, Nancy Keating, Anne Kirchoff, Michelle Kirschner, Ron Kline, Jessica MacIntyre, Molly Maher, Deborah Mayer, Janette Merrill, Timothy Mullett, Larissa Nekhlyudov, Frank Penedo, Mackenzi Pergolotti, Michael Roth, Tara Sanft, Alyssa Schatz, Ethan Schilling, Kathryn Schmitz, Lisa Schwartz, Emily Tonorezos

**Affiliations:** 1 Division of Cancer Control and Population Sciences, National Cancer Institute, 9609 Medical Center Drive, MSC 9712, Room 3E440, Bethesda, MD 20892-9762 USA; 2 Department of Veterans Affairs, National Oncology Program, Washington, DC USA; 3 Hematology/Oncology Service, Brooke Army Medical Center, Defense Health Agency, San Antonio, TX USA; 4 Uniformed Services University of Health Sciences, Bethesda, MD USA; 5 Keck School of Medicine, University of Southern California, Los Angeles, CA USA; 6 Children’s Hospital Los Angeles and USC Norris Comprehensive Cancer Center, Los Angeles, CA USA; 7 Department of Health Services Research, Peter MacCallum Cancer Centre, Melbourne, VIC Australia; 8 Australian Cancer Survivorship Centre, Peter MacCallum Cancer Centre, Melbourne, VIC Australia; 9 Sir Peter MacCallum Department of Oncology, University of Melbourne, Melbourne, VIC Australia; 10 Office of The Assistant Secretary of Defense for Health Affairs, Department of Defense, Washington, DC USA; 11 Department of Surgery, College of Medicine, University of Kentucky, Lexington, KY USA; 12 National Coalition for Cancer Survivorship, Silver Spring, MD USA; 13 Cancer Survivorship Advocate, Carolina Pediatric Therapy, Asheville, NC USA; 14 Division of Hematology/Oncology, University of Pittsburgh School of Medicine, Pittsburgh, PA USA; 15 Section of Hematology/Oncology, VA Pittsburgh Healthcare System, Pittsburgh, PA USA; 16 VA National TeleOncology, Durham, NC USA

**Keywords:** Survivor, Health system, Survivorship, Care quality

## Abstract

**Purpose:**

To develop United States (US) standards for survivorship care that informs (1) essential health system policy and process components and (2) evaluation of the quality of survivorship care.

**Methods:**

The National Cancer Institute and the Department of Veterans Affairs led a review to identify indicators of quality cancer survivorship care in the domains of health system policy, process, and evaluation/assessment. A series of three virtual consensus meetings with survivorship care and research experts and advocates was conducted to rate the importance of the indicators and refine the top indicators. The final set of standards was developed, including ten indicators in each domain.

**Results:**

Prioritized items were survivor-focused, including processes to both assess and manage physical, psychological, and social issues, and evaluation of patient outcomes and experiences. Specific indicators focused on developing a business model for sustaining survivorship care and collecting relevant business metrics (e.g., healthcare utilization, downstream revenue) to show value of survivorship care to health systems.

**Conclusions:**

The National Standards for Cancer Survivorship Care can be used by health systems to guide development of new survivorship care programs or services or to assess alignment and enhance services in existing survivorship programs. Given the variety of settings providing care to survivors, it is necessary for health systems to adapt these standards based on factors including age-specific needs, cancer types, treatments received, and health system resources.

**Implications for Cancer Survivors:**

With over 18 million cancer survivors in the United States, many of whom experience varied symptoms and unmet needs, it is essential for health systems to have a comprehensive strategy to provide ongoing care. The US National Standards for Survivorship Care should serve as a blueprint for what survivors and their families can anticipate after a cancer diagnosis to address their needs.

**Supplementary Information:**

The online version contains supplementary material available at 10.1007/s11764-024-01602-6.

## Introduction

A cancer survivor is any individual from the point of diagnosis through the balance of life [1]. There are over 18 million cancer survivors in the United States [2], and with advances in diagnostic and treatment capabilities and the aging population, this number is expected to grow. People with cancer have unique survivorship needs, including physical and psychological symptoms both during and after their treatment, risk for recurrence and subsequent cancers, and social needs. As a result, most survivors require long-term follow-up care.

Survivorship care is multifaceted, and recommendations have included surveillance for recurrence and new cancers, prevention and management of physical and psychosocial symptoms, and promoting healthy behaviors [3, 4]. While survivorship guidelines exist [5–13], the delivery of survivorship care, including what care is delivered, to whom it is delivered, and who delivers the care, varies greatly based on factors including care setting, geographical area, and personal resources. Survivorship care is often fragmented, depending on survivors to seek care from multiple providers without a coordinated system. This is further exacerbated by differing philosophies concerning when survivorship care should be delivered (e.g., post-treatment for those treated with curative intent versus post-diagnosis for anyone with a cancer diagnosis). Survivorship care for many people in the United States is suboptimal, leaving survivors with persistent symptoms, unmet needs, and lack of access to comprehensive care.

There have been several previous efforts to define survivorship care. The LIVESTRONG Essential Elements of Survivorship Care were developed in 2011 with the goal of building consensus in the survivorship community around how best to address the needs of post-treatment survivors [14]. The American College of Surgeons’ Commission on Cancer (CoC) Survivorship Standard 4.8, recently updated in 2019, defined requirements for CoC accredited programs [15]. The updated survivorship standards require a survivorship coordinator, a survivorship program documenting a minimum of three services offered each year to support patients, and a focus on enhancing existing and developing new services. This revised standard was an update from the 2016 survivorship standard that required documentation of a survivorship care plan for patients with early-stage cancer treated with curative intent [16]. In addition, the Quality of Survivorship Care Framework was developed to define the key components of quality survivorship care that are applicable to diverse populations of adult cancer survivors and was intended to inform clinical care, research, and policy [3].

Given that people with cancer are treated in diverse settings, including cancer centers, academic medical centers, and community sites, there is a need for developing a comprehensive set of national standards for health systems to provide quality survivorship care. The overall goal of this project was to build upon existing efforts to develop national standards for survivorship care that can be utilized by all healthcare systems to assess the quality of existing survivorship care and guide the development of new programs and services. Standards of care represent recommendations for health systems that apply to the patients they serve. Specifically, we sought to define standards for (1) essential health system policy and process components of survivorship care programs and (2) the evaluation of the quality of survivorship care.

## Methods

The Biden Cancer Moonshot, President Biden’s whole of government response to accelerate progress against cancer and end cancer as we know it, established a goal to develop standards for survivorship care. This project was led by the National Cancer Institute and United States Department of Veterans Affairs, in collaboration with several other Health and Human Services Agencies. Methods were adapted from a previous effort in Australia, where an online modified reactive Delphi survey was completed, followed by a consensus meeting of survivorship experts to inform the Victorian Quality Cancer Survivorship Framework [17].

### Key definitions

For the purpose of this project, we defined a cancer survivor as any individual from the time of diagnosis through the balance of life, diagnosed at any age or stage. We also adapted definitions from Lisy et al. for health system policy, health system process, and evaluation/assessment [17]. Health system policies were defined as principles and procedures guiding an organization’s capacity and structure to provide survivorship care; health system processes were an organization’s capacity to deliver care through its embedded practices and procedures; and evaluation/assessment were how to measure the impacts of survivorship care within an organization.

### Identification of possible indicators

A list of potential indicators in the three domains of health system policy, process, and evaluation/assessment were identified through a review of survivorship and cancer-specific guidelines, the CoC survivorship standard [15], existing survivorship quality frameworks [3, 17], US cancer control plans [18], and relevant literature. These resources were gathered based on the recommendations of the Task Force and subject matter experts.

### Subject matter expert consensus meetings

In 2023, three virtual meetings with survivorship subject matter experts were held to prioritize the most important and feasible indicators to include in the standards. The three meetings were iterative and invited subject matter experts included leading national and international experts in clinical survivorship care, survivorship research, implementation science, health policy, and survivor advocates. Subject matter experts were chosen based on their knowledge of the evidence related to survivorship care and/or their experience in providing care, informing health policy, and/or conducting survivorship care delivery research. We utilized a snowball approach to identify experts and accepted additional recommendations from invited experts, with the overall goal of collectively representing diverse perspectives and experiences related to survivorship. A total of 35 experts participated in the meetings. Additionally, these meetings were open for public viewing and attendees were able to submit comments and questions for consideration and comment.

Meeting 1 focused on providing background to the project, an open discussion among the experts, and individual polling where experts rated the importance of each possible survivorship indicator and identified other indicators for consideration in the next round. Importance was defined using the definition from Lisy and colleagues, as “a core component in achieving survivorship care and can be used to measure the quality of survivorship care” [17]. For the first meeting, experts were asked only to consider the importance of each indicator rather than also considering the feasibility of implementing and collecting this information. Experts could also suggest edits to the indicators. Questions from Meetings 1–3 can be found in the supplementary information (Appendix A).

Responses from the Meeting 1 poll were aggregated to identify those rated most important and those rated least important. Based on those results and suggestions from experts and public viewers on edits and additional indicators, an updated list of 15–20 indicators in each domain (policy, process, evaluation/assessment) was developed. Meeting 2 was then held one week later, where results from Meeting 1 were shared, including the indicators rated most important and those rated least important. Following was an open discussion of the results among the experts, including a discussion of feasibility. Experts were then asked to select the top 10 most important and feasible (to implement and/or collect) indicators within each domain; they could also suggest edits to the indicators.

Responses from Meeting 2 were then aggregated to identify the top 10 rated most important and feasible indicators in each domain. Results were shared with experts during Meeting 3, followed by an open discussion of the results. A final poll was conducted where experts were asked to suggest edits to the top 10 indicators in domain and to identify indicators that did not make the top 10 but should be considered for inclusion in the final standards.

Based on suggested edits and additions during the Meeting 3 poll and through refinement by the co-chairs, a final set of standards was developed that includes 10 indicators in health system policy, processes, and evaluation/assessment.

## Results

### Meeting 1 results

The poll for Meeting 1 included 18 indicators for health system policy, 33 indicators for processes, and 20 indicators for evaluation/assessment. Based on polling results, the *policy* indicators rated highest importance were a policy requiring establishment of a survivorship program, outlining a team of multidisciplinary health professionals included in the survivorship program, collection of data on survivors’ experience of care and patient-reported outcomes, stratifying survivors to appropriate models of care, provision of support services to survivors based on needs, consideration of approach and timing of transitions in survivorship care, training for healthcare providers, and designation of an organizational survivorship care leader. The *policy* indicators rated lowest importance were a policy for documenting survivorship care reporting requirements to a government agency, public reporting and dissemination of survivorship outcomes, documenting a minimum of three services offered each year to support patients and survivors, and providing access to prescription produce programs using existing systems/programs. The *process* indicators rated highest importance were assessment of emotional and psychological effects of cancer and its treatment, physical effects during and following cancer treatment, risk of recurrence or new cancers, practical and social effects (e.g., financial challenges), lifestyle behaviors, and provided with treatment, referrals, and advice to manage physical, emotional, and social effects. The *process* indicators rated lowest importance were providing the opportunity for participating in research including clinical trials, providing support or referrals for other medical or chronic conditions that are non-cancer related, providing access to advice on vaccinations, providing a meeting to plan survivorship care at the time of diagnosis, providing medically tailored food and nutrition services, providing information and access to complementary health services to support overall health and well-being, and providing a consultation with palliative care. For *evaluation*, indicators rated highest importance were survivors’ patient-reported outcomes, quality of life, patient-reported experiences of care, return to work, and functional capacity. The *evaluation* indicators rated lowest importance were overall cost of survivorship care to the health system, number of survivors provided with a survivorship care plan, health professionals’ view of survivorship care, survivors’ hospital admissions, number of referrals made for survivors, and number of primary care providers who are sent a survivorship care plan.

### Meeting 2 results

The poll for Meeting 2 included the top 15 indicators for health system policy, 20 indicators for processes, and 20 indicators for evaluation/assessment. The indicators that were ranked in the top 10 for *policy* included a policy that requires establishment or existence of a survivorship program either on-site or by referral; that describes framework for the provision of survivorship care informed by relevant survivorship guidelines (e.g., ASCO, NCCN, ACS), on stratifying survivors to appropriate models of care; that designates an organizational survivorship care lead who evaluates compliance with standards, has senior role in healthcare system, and includes succession plan for the role, outlining team of multidisciplinary health professionals included in survivorship program; and that considers approach and timing of transitions in survivorship care (e.g., pediatric to adult, acute to primary care, oncology team to survivorship team), for the provision of support services to survivors with special needs and from diverse backgrounds (e.g., navigators, interpreters), for training healthcare providers to deliver survivorship care, for collection of data on survivors’ experience of survivorship care and patient-reported outcomes, and for outlining business case/plan with funding allocated for survivorship care (to include budget). *Policy* indicators not ranked in the top 10 included outlining the provision of needs assessment tools for survivors at certain time points post-treatment; requiring survivorship-focused information available in other languages or different formats for low-literacy readers; outlining the role of survivors in design, evaluation, and reporting of progress; documenting survivorship care reporting requirements to relevant organizational executive committee; and collecting data on caregivers’ experiences of survivorship care.

For *processes*, the indicators that were ranked in the top 10 were that cancer survivors were provided access to a survivorship program which addresses the needs of cancer survivors either on-site or by referral; assessed for physical effects during and following cancer treatment, including monitoring for late effects and chronic conditions, and provided with treatment and/or referrals; assessed for emotional and psychological effects of cancer and its treatment and provided with treatment and/or referrals; assessed for practical and social effects of cancer and its treatment (e.g., relationship difficulties, financial challenges, education and employment/return to work) and provided with resources and/or referrals, provided with recommendations regarding surveillance for recurrent or new cancers; assessed for their risk of recurrence or new cancers, including family history and genetic testing; assessed for lifestyle behaviors with recommended management and/or provided with appropriate referral (e.g., smoking cessation, promoting physical activity); provided with access to allied health services (e.g., nutrition, physical therapy, sexual health, fertility services, rehabilitation, dental and podiatry services); provided with access to specialty care services to manage potential late effects (e.g., cardiology); assessed for financial hardship/toxicity and provided with resources and support; and provided with care planning conversations including coordination of care with primary care provider and/or other multidisciplinary health professionals involved in their care. The *process* indicators not ranked in the top 10 were providing care consistent with their goals, providing access to care to manage fertility and reproductive concerns, providing access to age-specific survivorship care, providing access to primary care services, providing access to age- and gender-appropriate cancer screening or referrals to appropriate screening services, and providing access to tobacco cessation services.

In the domain of evaluation/assessment, indicators ranked in the top 10 were survivors’ and caregivers’ patient-reported outcomes, including quality of life, functional capacity, survival rates (1, 5, and 10 years), experiences of care, return to work, rate of recurrence and new cancers, number and characteristics of survivors lost to follow-up, number of survivors with subsequent chronic condition, rate of survivor service referrals and completions, and relevant business metrics to show return on investment of survivorship care to the healthcare system. *Evaluation* indicators that were not ranked in the top 10 were collecting data on the number of health professionals trained to provide survivorship care, the number of survivors who have their needs assessed at certain times post-treatment, overall cost of care to survivors and caregivers, survivors’ emergency care and urgent care utilization, number of survivors stratified to different models of care, and oncology providers’ view of the role of nurses and advanced practice providers in survivorship care.

### Meeting 3 results

The poll for Meeting 3 included an updated list of the top 10 highest-rated indicators in each domain, as well as a list of the indicators that did not make the top 10. Experts were asked to suggest edits, including suggestions to combine indicators, as well as identify lower-rated indicators to consider for inclusion. Lower-rated indicators that were identified by at least 40% of experts to consider for inclusion were then either combined with other indicators or added to the final set. For health system policy, this included a policy outlining the role of survivors in design, evaluation, and reporting of progress. For processes, this included providing care that is consistent with goals, and consideration of age-specific care. For evaluation/assessment, this included the number of health professionals trained to provide survivorship care.

### Final cancer survivorship standards

The National Cancer Standards for Survivorship Care are presented below and in Table 1 and include the top 10 indicators in each of the three domains of health system policy, process, and evaluation/assessment.

**Table 1:** National standards for cancer survivorship care

Health system policy	Health system processes	Health system evaluation/assessment
The organization has a policy that includes…	Cancer survivors are…	The organization has a process to collect data on…
Establishment or existence of a survivorship program either on-site, through telehealth, or by referral	Provided with access and referral to a survivorship program that addresses the needs of cancer survivors either on-site, through telehealth, or by referral	Survivors’ patient-reported outcomes, including quality of life, and experiences of survivorship care
A framework for the provision of survivorship care informed by survivor stakeholders and relevant survivorship guidelines (e.g., American Society of Clinical Oncology, National Comprehensive Cancer Network, Children’s Oncology Group)	Assessed at multiple points in their follow-up care for physical effects during and following cancer treatment, including monitoring for late effects and chronic conditions, and provided with treatment and/or referrals	Survivors’ functional capacity
A description of multidisciplinary care, including each team member’s specific roles and responsibilities and workflow(s) for referrals to team members	Provided with access to appropriate specialty care services to manage potential late effects (e.g., cardiovascular issues) either on-site, through telehealth, or by referral	Survivors’ return to previous participation in paid and unpaid work/ school/ productive activities of living
An overview of how to stratify and refer survivors to appropriate models of care based on age, treatments, and risk factors	Assessed at multiple points in their follow-up care for emotional and psychological effects of cancer and its treatment and provided with treatment and/or referrals	Survival rates (1, 5, and 10 years) from the time of diagnosis
Description of the approach and timing of transitions in survivorship care and shared care (e.g., pediatric to adult providers and settings, oncology team to survivorship team and/or primary care) and efforts to prevent/mitigate loss to follow-up care	Assessed for practical and social effects of cancer and its treatment (e.g., social risks, health-related social needs, education and employment/return to work or school) and provided with resources and/or referrals	Rate of recurrence
An outline for the provision of information for support services (e.g., navigators, social work, interpreters) for survivors based on their needs (including but not limited to health, insurance, and financial literacy, disability status), including survivors from diverse and underserved backgrounds	Assessed for their risk of recurrence or new cancers, including family history and genetic testing, and provided with recommendations and referrals regarding surveillance for recurrence or new cancers	Rate of subsequent cancers
Identification of an executive-level survivorship care lead (with succession plan) whose role is to ensure compliance with standards, with reporting to an appropriate executive committee	Assessed for lifestyle behaviors and provided with recommended strategies for management and appropriate referrals or education as needed (e.g., smoking cessation, diet/nutrition counseling, promoting physical activity)	Number and relevant characteristics (demographics, clinical factors) of survivors lost to follow-up
Collection of longitudinal data on survivors’ experience of survivorship care and patient-reported outcomes	Provided with access and referrals to appropriate supportive health services (e.g., nutrition, occupational and physical therapy, rehabilitation, sexual health, fertility services, dental and podiatry services)	Caregivers’ experiences and unmet needs
Requirements and methods for training healthcare providers (either on-site or through an external training program) to deliver survivorship care within their scope of practice	Assessed for financial hardship/toxicity and concerns regarding insurance coverage, and provided with resources and support as needed	Number of health professionals trained to provide survivorship care
A business case/plan, including budget, with funding allocated for survivorship care	Engaged in the care planning process including discussion of shared goals of care, advanced care planning, and coordination of care with providers and services (e.g., primary care provider, other health professionals, and community-based services) as needed	Relevant business metrics to show return on investment of survivorship care to the healthcare system (e.g., healthcare utilization, rate of referrals and completion, downstream revenue)

Note: This table represents the final National Standards for Cancer Survivorship Care, intended to be utilized to assess survivorship programs as they align to each of the indicators and guide development of new programs

### Health system policy

The organization has a policy that includes:

- Establishment or existence of a survivorship program either on-site, through telehealth, or by referral
- A framework for the provision of survivorship care informed by survivor stakeholders and relevant survivorship guidelines (e.g., American Society of Clinical Oncology, National Comprehensive Cancer Network, Children’s Oncology Group)
- A description of multidisciplinary care, including each team member’s specific roles and responsibilities and workflow(s) for referrals to team members
- An overview of how to stratify and refer survivors to appropriate models of care based on age, treatments, and risk factors
- Description of the approach and timing of transitions in survivorship care and shared care (e.g., pediatric to adult providers and settings, oncology team to survivorship team and/or primary care) and efforts to prevent/mitigate loss to follow-up care
- An outline for the provision of information for support services (e.g., navigators, social work, interpreters) for survivors based on their needs (including but not limited to health, insurance, and financial literacy, disability status), including survivors from diverse and underserved backgrounds
- Identification of an executive-level survivorship care lead (with succession plan) whose role is to ensure compliance with standards, with reporting to an appropriate executive committee
- Collection of longitudinal data on survivors’ experience of survivorship care and patient-reported outcomes
- Requirements and methods for training healthcare providers (either on-site or through an external training program) to deliver survivorship care within their scope of practice
- A business case/plan, including budget, with funding allocated for survivorship care

### Health system processes

Cancer survivors are…

- Provided with access and referral to a survivorship program that addresses the needs of cancer survivors either on-site, through telehealth, or by referral
- Assessed at multiple points in their follow-up care for physical effects during and following cancer treatment, including monitoring for late effects and chronic conditions, and provided with treatment and/or referrals
- Provided with access to appropriate specialty care services to manage potential late effects (e.g., cardiovascular issues) either on-site, through telehealth, or by referral
- Assessed at multiple points in their follow-up care for emotional and psychological effects of cancer and its treatment and provided with treatment and/or referrals
- Assessed for practical and social effects of cancer and its treatment (e.g., social risks, health-related social needs, education and employment/return to work or school) and provided with resources and/or referrals
- Assessed for their risk of recurrence or new cancers, including family history and genetic testing, and provided with recommendations and referrals regarding surveillance for recurrence or new cancers
- Assessed for lifestyle behaviors and provided with recommended strategies for management and appropriate referrals or education as needed (e.g., smoking cessation, diet/nutrition counseling, promoting physical activity)
- Provided with access and referrals to appropriate supportive health services (e.g., nutrition, occupational and physical therapy, rehabilitation, sexual health, fertility services, dental and podiatry services)
- Assessed for financial hardship/toxicity and concerns regarding insurance coverage, and provided with resources and support as needed
- Engaged in the care planning process including discussion of shared goals of care, advanced care planning, and coordination of care with providers and services (e.g., primary care provider, other health professionals, and community-based services) as needed

### Health system evaluation/assessment

The organization has a process to collect data on…

- Survivors’ patient-reported outcomes, including quality of life, and experiences of survivorship care
- Survivors’ functional capacity
- Survivors' return to previous participation in paid and unpaid work/ school/ productive activities of living
- Survival rates (1, 5, and 10 years) from the time of diagnosis
- Rate of recurrence
- Rate of subsequent cancers
- Number and relevant characteristics (demographics, clinical factors) of survivors lost to follow-up
- Caregivers’ experiences and unmet needs
- Number of health professionals trained to provide survivorship care
- Relevant business metrics to show return on investment of survivorship care to the healthcare system (e.g., healthcare utilization, rate of referrals and completion, downstream revenue)

## Discussion

Efforts to advance survivorship care have largely been focused on development of evidence-based guidelines and defining the key components of quality survivorship care. Survivorship care services vary greatly among cancer centers and in the community [19]. Given the growing population of survivors treated in a variety of care settings, it is essential to define a standard for health systems to care for survivors. This current effort aimed to address this gap by developing national standards to define and prioritize key health system policy, process, and evaluation/assessment indicators. While evidence-based guidelines inform provider practices [5], and the Nekhlyudov framework identifies key components to survivorship care [3], the standards presented herein build on this previous work. They are intended to be utilized to assess survivorship programs within a health system or organization to address the comprehensive needs of cancer survivors during and after treatment.

The methods for this project were adapted from the Victorian Quality Cancer Survivorship Framework [17], and the resulting indicators differed in several ways. First, consensus meeting discussions included the need to de-emphasize survivorship care plan documentation, given limited evidence on improving survivor outcomes [20]. In addition, these standards incorporate all modalities to offer survivorship care services, including telehealth. Experts also identified the need to emphasize support for care transitions across the continuum from diagnosis forward, to include a policy requiring training of healthcare professionals to deliver survivorship care, and subsequently to assess the number of providers trained. Though it was recognized that assessment of survival would be challenging, experts also recognized the need to include and aspire to collect long-term survival data after diagnosis with cancer (1, 5, and 10 years). Finally, US standards include a policy to develop a business case/plan with funding allocated for survivorship care, as well as relevant business metrics to show return on investment for survivorship care. Experts in the consensus meeting stressed the need for a sustainable business model for delivering survivorship care services that is evaluated longitudinally using appropriate metrics including (but not limited to) overall healthcare utilization, rate of referrals and completion, and downstream revenue to the organization or healthcare system. If organizations are to provide quality care for the growing number of survivors, it will be critical to show financial impacts for the healthcare system. An additional process indicator focused specifically on assessing and mitigating survivors’ financial hardship and concerns regarding insurance coverage. This is not surprising given the high proportion of cancer survivors who reporting experiencing financial challenges in the United States [21].

Consensus meetings also discussed considerations that health systems should take when implementing these standards. In the area of health system policy, experts and advocates noted that these indicators could be combined into one survivorship policy that informs care system-wide. One advantage of separating these indicators, however, is that key informants or stakeholders could be included in development or writing the individual policies. Furthermore, the impact of separate policy changes could be evaluated individually. Additionally, several experts noted that process indicators related to physical, psychological, and social impacts of cancer and its treatment should go beyond only assessment for late effects and should include management and specialty referral, as indicated. Experts and advocates also noted that in the area of evaluation/assessment, validated measures should be utilized whenever possible. While this process was not intended to endorse specific measures or tools, participants emphasized an expectation that validated, patient-centered measures would be used. Finally, health systems should ensure consent has been obtained from survivors and caregivers before assessment.

The final set of standards represents input from survivorship experts and advocates and can be implemented in a variety of settings. A key next step in this work is to implement the standards within healthcare systems that are developing new survivorship programs or have existing programs or services. Healthcare systems that provide care for people after a cancer diagnosis, including but not limited to cancer centers, may use these standards to assess organizational alignment and enhance their survivorship care services. After aligning with the standards, there will be a need to evaluate for feasibility, potential for sustainability, and impact on survivor outcomes. It is important to note that use of these standards by health systems is voluntary, and components of care may or may not be covered by public or private health insurance.

There are several key considerations when implementing these survivorship standards to inform survivorship program development or assess alignment with the indicators. First, while these standards were intended to inform health systems caring for cancer survivors diagnosed at any age, with any cancer, and at any stage, there is a need to tailor care services based on specific factors, including age, setting, and specific cancer types and treatment. For example, while we utilized the NCI recognized definition of a cancer survivor from the time of diagnosis through the balance of life [1], the standards could also be applied to post-treatment survivors. In addition, survivors of pediatric cancers diagnosed between birth and 15 years may have markedly different needs from survivors of adolescent and young adult (AYA) or older adult survivors [22, 23]. For patients diagnosed as young children, survivorship care and research are already highly developed with effective, existing clinical models that constitute “standard of care” for this population. Indeed, in many ways pediatric survivorship care and research have inspired adult-focused efforts. But in pediatric cancer survivorship, particularly for well-established treatments, the evidence base for late effects and their trajectory is well-established and comprehensive. For most childhood cancer survivors, recommended survivorship care for late effects monitoring and management is generally annual, lifelong follow-up, including transition to adult-focused care during young adulthood [24]. There is still significant work to be done to improve transition services and outcomes [25], which is one area where the standards could be very informative. Existing guidelines for childhood cancer survivorship care should continue to be utilized [13].

For survivors of AYA cancers (diagnosed between 15 and 39 years old), health systems and providers must pay close attention to the unique needs of this population. While the components of their survivorship care may be similar to older adults, AYAs are particularly vulnerable to adverse impacts of cancer on education, career development, work, financial status, and psychosocial needs. Fertility is a particular medical concern for this cancer population. Although a separate, parallel set of survivorship standards for survivors of AYA cancer may not be necessary, it is crucial these standards be applied in a manner responsive to their needs. For older adult survivors, geriatric assessment and focused provider training could be incorporated to address the unique considerations of older adults with a history of cancer [26]. Overall, the standards should be used as a guide for health systems to adapt based on the known needs of populations served.

Though the results of this work represent national standards for survivorship care, these standards can also be utilized to inform survivorship research. The NCI has supported key efforts in survivorship care, including funding opportunities focused on addressing primary care for cancer survivors [27] and optimizing survivorship care for survivors transitioning between oncology and non-oncology providers [28]. A challenge in delivering and evaluating survivorship care, however, is that there has not been an accepted national standard. This project represents consensus agreement of national experts on essential policy, process, and evaluation components to survivorship care that health systems should utilize based on the available evidence. Rigorous evaluation of the implementation and outcomes of these standards will be critical to show continued value to follow-up care for people after a cancer diagnosis [29]. Additionally, the evaluation/assessment indicators of these standards may be used as meaningful endpoints for survivorship care interventions to show impact on survivor and health system outcomes.

These standards represent a key foundation for improving the delivery of survivorship care across the United States; however, there are limitations to this work. First, though a robust review was conducted to identify potential indicators, it is possible that specific literature may have been inadvertently missed. In addition, the consensus meetings represented a diverse panel of experts and survivor advocates who provided feedback and input. It is possible, however, that some perspectives were not represented in the expert group. One important perspective missing is that of a healthcare business administrator, who will be essential in converting these standards to implementation of survivorship programs. A key next step in this work will be collaboration with healthcare administrators and payors to translate these recommendations into action. The selection of experts also may lead to limitations in the prioritization of indicators. Future empirical support is needed to provide evidence of the outcomes of implementing the standards.

### Supplementary Information

Below is the link to the electronic supplementary material.

Supplementary file1 (DOCX 25 KB)

## Data Availability

No datasets were generated or analyzed during the current study. This study has not been previously presented.

## Declarations

### Disclaimers

The view(s) expressed herein are those of the author(s) and do not reflect the official policy or position of the Defense Health Agency, Department of Defense, or the US Government. Mention of trade names, commercial products, or organizations does not imply endorsement by Department of Defense or the US Government. The views expressed herein are those of the authors and do not reflect the official policy or position of the Department of Veterans Affairs or the US Government.
