# Correction: Developing national cancer survivorship standards to inform quality of care in the United States using a consensus approach

**DOI:** 10.1007/s11764-024-01618-y

**Published:** 2024-06-15

**Authors:** Michelle A. Mollica, Gina McWhirter, Emily Tonorezos, Joshua Fenderson, David R. Freyer, Michael Jefford, Christopher J. Luevano, Timothy Mullett, Shelley Fuld Nasso, Ethan Schilling, Vida Almario Passero, the National Cancer Survivorship Standards Subject Matter Expert Group

**Affiliations:** 1 Division of Cancer Control and Population Sciences, National Cancer Institute, 9609 Medical Center Drive, MSC 9712, Room 3E440, Bethesda, MD 20892-9762 USA; 2 Department of Veterans Affairs, National Oncology Program, Washington, DC USA; 3 Hematology/Oncology Service, Brooke Army Medical Center, Defense Health Agency, San Antonio, TX USA; 4 Uniformed Services University of Health Sciences, Bethesda, MD USA; 5 Keck School of Medicine, University of Southern California, Los Angeles, CA USA; 6 Children’s Hospital Los Angeles and USC Norris Comprehensive Cancer Center, Los Angeles, CA USA; 7 Department of Health Services Research, Peter MacCallum Cancer Centre, Melbourne, VIC Australia; 8 Australian Cancer Survivorship Centre, Peter MacCallum Cancer Centre, Melbourne, VIC Australia; 9 Sir Peter MacCallum Department of Oncology, University of Melbourne, Melbourne, VIC Australia; 10 Office of The Assistant Secretary of Defense for Health Affairs, Department of Defense, Washington, DC USA; 11 Department of Surgery, College of Medicine, University of Kentucky, Lexington, KY USA; 12 National Coalition for Cancer Survivorship, Silver Spring, MD USA; 13 Cancer Survivorship Advocate, Carolina Pediatric Therapy, Asheville, NC USA; 14 Division of Hematology/Oncology, University of Pittsburgh School of Medicine, Pittsburgh, PA USA; 15 Section of Hematology/Oncology, VA Pittsburgh Healthcare System, Pittsburgh, PA USA; 16 VA National TeleOncology, Durham, NC USA

**Correction: Journal of Cancer Survivorship**

10.1007/s11764-024-01602-6

In the original article, the following author name is missing under the acknowledgement section (National Survivorship Standards Subject Matter Expert Group)

Expert: Lawrence Shulman, MD

Organization: University of Pennsylvania

This has been corrected in the paper.

