# Supplementary material for: Developing national cancer survivorship standards to inform quality of care in the United States using a consensus approach
Source: J Cancer Surviv. 2024 May 13;18(4):1190–9. doi: 10.1007/s11764-024-01602-6 (PMC11324674; doi:10.1007/s11764-024-01602-6)
Supplement: Supplementary file 1 — Supplementary file1 (DOCX 25 KB) [file 11764_2024_1602_MOESM1_ESM.docx]

Appendix A.

**Meeting 1 Questions**

Please rate the importance of each indicator as not at all important, not important, somewhat important, important, and very important.

As you are rating, please note that for the purpose of these questions, importance is defined as being a “core component in achieving quality survivorship care and can be used to measure the quality of survivorship care” (Lisy et al.). For this step, please consider the importance of each indicator in optimal survivorship care rather than considering practical aspects or feasibility of collecting the data.

**Section 1. Health system policy**

**How important are each of the following indicators in achieving quality survivorship care?**

**The organization has a policy that…**

- describes framework for the provision of survivorship care
- requires establishment or existence of a survivorship program either on-site or by referral
- outlines team of multidisciplinary health professionals included in survivorship program
- collects data on survivors' experience of survivorship care (e.g., satisfaction)
- outlines role of survivors in design, evaluation, and reporting of progress
- stratifies survivors to appropriate models of care
- outlines provision of needs assessment tools for survivors at certain time points post-treatment
- provides support services to survivors with special needs and from diverse backgrounds (e.g., navigators, interpreters)
- considers transitions in survivorship care (e.g., pediatric to adult, acute to primary care)
- documents survivorship care reporting requirements to relevant organizational executive committee
- requires survivorship-focused information available in other languages or different format for patients with low literacy
- collects data on caregivers' experiences of survivorship care
- documents survivorship care reporting requirements to a government agency
- requires public reporting and dissemination of survivorship outcomes
- trains healthcare providers to deliver survivorship care
- documents a minimum of 3 services offered each year to support patients and survivors
- designates an organizational survivorship care lead who evaluates compliance with standards and has senior role in healthcare system
- provides access to prescription produce program using existing systems/programs

**Open question: Are there any indicators for health system policies that are missing and should be added for consideration?**

**Section 2. Health system processes**

**How important are each of the following indicators in achieving quality survivorship care?**

**Cancer survivors are assessed for….**

- emotional and psychological effects of cancer and its treatment
- physical effects during and following cancer treatment, including monitoring for late effects
- their risk of recurrence or new cancers, including family history
- practical and social effects of cancer and its treatment (e.g., relationship difficulties, financial challenges, education and employment/return to work)
- lifestyle behaviors with recommended management or provided with an appropriate referral (e.g., smoking cessation)
- their self-management skills and appropriately stratified according to their ability to self-manage with support
- adherence to recommended strategies to manage consequences of cancer and its treatment

**How important are each of the following indicators in achieving quality survivorship care?**

**Cancer survivors are provided with…**

- stratification to appropriate models of care based on factors such as current needs and predicted risks
- treatment, referrals, and advice to manage physical, psychological effects, and/or chronic medical conditions
- access to a survivorship program which addresses the needs of cancer survivors either on-site or by referral
- care planning conversations including a survivorship care plan that is shared with primary care provider and/or other multidisciplinary health professionals involved in their care
- recommendations regarding surveillance for recurrent or new cancers provided with treatment or referrals to psychosocial effects
- recommendations to reduce the risk of physical and psychological effects (e.g., weight loss, exercise)
- care which is consistent with their goals
- access to allied health services (e.g., nutrition, physical therapy, sexual health, rehabilitation, dental and podiatry services)
- access to education and resources about the post-treatment phase to meet individuals' needs, understanding, and health literacy
- access to primary care services
- access to telehealth services and remote surveillance programs where appropriate
- opportunities to participate in research projects including clinical trials
- provided access to specialty care services to manage potential late effects (e.g., cardiology)
- access to age- and gender-appropriate cancer screening or referrals to appropriate screening services
- access to education and resources for their caregivers about the post-treatment phase which meets their needs, understanding, and health literacy
- support or referrals for other medical or chronic conditions which are non-cancer related
- referrals for genetic testing as necessary following primary treatment
- access to advice on vaccinations
- services to coordinate care (e.g., navigation)
- meeting to plan survivorship care at time of diagnosis
- access to smoking cessation services
- medically tailored food and nutrition services
- information and access to complementary healthcare services to support overall health and well-being (e.g., mindfulness, meditation, yoga)
- resources on how to talk to children about cancer based on their developmental stage – there are a lot of existing resources that just need to be utilized systematically to support families
- resources and support to mitigate financial hardship/ toxicity
- consultation with palliative care at diagnosis as appropriate

**Open question: Are there any indicators for health system processes that are missing and should be added for consideration?**

**Section 3. Health system evaluation**

**How important are each of the following indicators in achieving quality survivorship care?**

**The organization has a process to collect data on…**

- survivors' patient-reported outcomes
- survivors' quality of life
- survival rates (1 and 5-yr)
- recurrence rates
- survivors' patient-reported experiences of care
- survivors' return to previous functioning (e.g., work)
- survivors' functional capacity
- diagnosis of new cancers for survivors
- caregivers' quality of life
- number of survivors provided with a survivorship care plan
- overall cost of care to survivors and caregivers
- overall cost of survivorship care to the health system
- number/proportion of survivors who have their needs assessed at a certain time point post-treatment
- number/proportion of primary care providers provided with a survivorship care plan
- number/proportion of survivors stratified to different models of care
- number/proportion of health professionals trained to provide survivorship care
- number/proportion and characteristics of survivors lost to follow-up
- health professionals' views of survivorship care
- survivors' hospital admissions
- number of referrals made for survivors

**Open question: Are there any indicators for health system evaluation that are missing and should be added for consideration?**

**Meeting 2 Questions**

**Please review the following list of indicators and identify the 10 most *important and feasible* indicators (to implement and/or collect) within each domain:**

**Question 1: Health system policies (select 10)**

**The organization has a:**

1. policy that describes framework for the provision of survivorship care informed by relevant survivorship guidelines (e.g., ASCO, NCCN, ACS)
2. policy that requires establishment or existence of a survivorship program either on-site or by referral
3. policy outlining team of multidisciplinary health professionals included in survivorship program
4. policy for collection of data on survivors' experience of survivorship care and patient-reported outcomes
5. policy that outlines role of survivors in design, evaluation, and reporting of progress
6. policy on stratifying survivors to appropriate models of care
7. policy outlining the provision of needs assessment tools for survivors at certain time points post-treatment
8. policy for the provision of support services to survivors with special needs and from diverse backgrounds (e.g., navigators, interpreters)
9. policy that considers approach and timing of transitions in survivorship care (e.g., pediatric to adult, acute to primary care, oncology team to survivorship team)
10. policy that document survivorship care reporting requirements to relevant organizational executive committee
11. policy that requires survivorship-focused information available in other languages or different format for low literacy readers
12. policy for collection of data on caregivers' experiences of survivorship care
13. policy for training healthcare providers to deliver survivorship care
14. policy that designates an organizational survivorship care lead who evaluates compliance with standards and has senior role in healthcare system
15. policy outlining business case/plan with funding allocated for survivorship care (to include budget)

**Question 2: Health system processes (select 10)**

**Cancer survivors are…**

1. assessed for emotional and psychological effects of cancer and its treatment
2. assessed for physical effects during and following cancer treatment, including monitoring for late effects
3. assessed for their risk of recurrence or new cancers, including family history and genetic testing
4. assessed for practical and social effects of cancer and its treatment (e.g., relationship difficulties, financial challenges, education and employment/return to work)
5. assessed for lifestyle behaviors with recommended management or provided with an appropriate referral
6. provided with treatment, referrals, and advice to manage physical, psychological effects, and/or chronic medical conditions
7. provided with access to a survivorship program which addresses the needs of cancer survivors either on-site or by referral
8. provided with recommendations regarding surveillance for recurrent or new cancers
9. provided with recommendations to reduce the risk of physical and psychological effects (e.g., weight loss, exercise)
10. provided with care which is consistent with their goals
11. provided with access to allied health services (e.g., nutrition, physical therapy, sexual health, rehabilitation, dental and podiatry services)
12. provided with access to education and resources about the post-treatment phase to meet individuals' needs, understanding, and health literacy
13. provided with access to primary care services
14. Provided with care planning conversations including a survivorship care plan that is shared with primary care provider and/or other multidisciplinary health professionals involved in their care
15. provided with access to specialty care services to manage potential late effects (e.g., cardiology)
16. provided with access to age- and gender-appropriate cancer screening or referrals to appropriate screening services
17. provided with resources and support to mitigate financial hardship/ toxicity
18. provided with access to care to manage fertility and reproductive concerns
19. provided with access to tobacco cessation services
20. provided with access to age-specific survivorship care (e.g., pediatric, geriatric expertise)

**Question 3: Health system evaluation/assessment (select 10)**

**The organization has a process to collect data on…**

1. survivors' patient-reported outcomes
2. survivors' quality of life
3. survival rates (1, 5, and 10-yr)
4. recurrence rates
5. survivors' patient-reported experiences of care
6. survivors' return to work
7. survivors' functional capacity
8. diagnosis of new cancers for survivors
9. caregivers' quality of life
10. overall cost of care to survivors and caregivers
11. number of survivors who have their needs assessed at certain time(s) post-treatment
12. number of survivors stratified to different models of care
13. number of health professionals trained to provide survivorship care
14. number and characteristics of survivors lost to follow-up
15. number of referrals made for survivors
16. oncology providers’ view of the role of nurses and advanced practice providers (APPs) in survivorship care
17. rate of survivor service referrals and completions
18. number of survivors with subsequent chronic conditions
19. survivors’ emergent care and urgent care utilization
20. relevant business metrics to show return on investment of survivorship care to the healthcare system

**Meeting 3 Questions**

**Note- all of these questions are optional**

**Health System Policy**

**Question 1. Please use the box below each indicator to suggest edits to the top 10 indicators in health system policy.**

**(Please have an open text box under each indicator that is optional)**

**The organization has a….**

1. policy that requires establishment or existence of a survivorship program either on-site or by referral
2. policy that describes framework for the provision of survivorship care informed by relevant survivorship guidelines (e.g., ASCO, NCCN, ACS)
3. policy on stratifying survivors to appropriate models of care
4. policy that designates an organizational survivorship care lead who evaluates compliance with standards and has senior role in healthcare system and includes succession plan for lead
5. policy outlining team of multidisciplinary health professionals included in survivorship program
6. policy that considers approach and timing of transitions in survivorship care (e.g., pediatric to adult, acute to primary care, oncology team to survivorship team)
7. policy for the provision of support services to survivors with special needs (including but not limited to health, insurance, and financial literacy) and from diverse backgrounds (e.g., navigators, interpreters)
8. policy for training healthcare providers to deliver survivorship care
9. policy for collection of data on survivors' experience of survivorship care and patient-reported outcomes
10. policy outlining business case/plan with funding allocated for survivorship care (to include budget)

**Policy**

**Question 2. Please check the box next to an indicator below if you would like it to be considered for inclusion in health system policies:**

**(Check all that apply and optional)**

- policy outlining the provision of needs assessment tools for survivors at certain time points post-treatment
- policy that requires survivorship-focused information available in other languages or different format for low literacy readers
- policy that outlines role of survivors in design, evaluation, and reporting of progress
- policy that document survivorship care reporting requirements to relevant organizational executive committee
- policy for collection of data on caregivers' experiences of survivorship care

**Health System Processes**

**Question 3. Please use the box below each indicator to suggest edits to the top 10 indicators in health system processes:**

**(Please have an open text box under each indicator that is optional)**

**Cancer survivors are…**

1. provided with access to a survivorship program which addresses the needs of cancer survivors either on-site or by referral
2. assessed for physical effects during and following cancer treatment, including monitoring for late effects and chronic conditions, and provided with treatment and/or referrals
3. assessed for emotional and psychological effects of cancer and its treatment and provided with treatment and/or referrals
4. assessed for practical and social effects of cancer and its treatment (e.g., relationship difficulties, financial challenges, education and employment/return to work) and provided with resources and/or referrals
5. provided with recommendations regarding surveillance for recurrent or new cancers and assessed for their risk of recurrence or new cancers, including family history and genetic testing
6. assessed for lifestyle behaviors with recommended management and/or provided with appropriate referral (e.g., smoking cessation, promoting physical activity)
7. provided with access to allied health services (e.g., nutrition, physical therapy, sexual health, fertility services, rehabilitation, dental and podiatry services)
8. provided with access to specialty care services to manage potential late effects (e.g., cardiology)
9. assessed for financial hardship/toxicity and provided with resources and support
10. provided with care planning conversations including coordination of care with primary care provider and/or other multidisciplinary health professionals involved in their care

**Question 4. Please check the box next to an indicator below if you would like it to be considered for inclusion in health system processes:**

**(Check all that apply and optional)**

- provided with care which is consistent with their goals
- provided with access to education and resources about the post-treatment phase to meet individuals' needs, understanding, and health literacy
- provided with access to care to manage fertility and reproductive concerns
- provided with access to age-specific survivorship care (e.g., pediatric, geriatric expertise
- provided with access to primary care services
- provided with access to age- and gender-appropriate cancer screening or referrals to appropriate screening services
- provided with access to tobacco cessation services

**Health System Evaluation**

**Question 5. Please use the box below each indicator to suggest edits to the top 10 indicators in health system evaluation:**

**(Please have an open text box under each indicator that is optional)**

**The organization has a process to collect data on…**

1. caregivers’ and survivors' patient-reported outcomes, including quality of life
2. survivors' functional capacity
3. survival rates (1, 5, and 10-yr)
4. survivors' experiences of care
5. survivors' return to work
6. rate of recurrence and new cancers
7. number and characteristics of survivors lost to follow-up
8. number of survivors with subsequent chronic conditions
9. rate of survivor service referrals and completions
10. relevant business metrics to show return on investment of survivorship care to the healthcare system

**Question 6. Please check the box next to an indicator below if you would like it to be considered for inclusion in health system evaluation/assessment:**

**(Check all that apply and optional)**

- number of health professionals trained to provide survivorship care
- number of survivors who have their needs assessed at certain time(s) post-treatment
- overall cost of care to survivors and caregivers
- survivors’ emergency care and urgent care utilization
- caregivers' quality of life
- number of survivors stratified to different models of care
- oncology providers’ view of the role of nurses and advanced practice providers (APPs) in survivorship care

**Question 7. Please use the text box below to add any additional comments:**
